# Comparative Analysis of Two Measurement Modalities for Ex Vivo Analysis of Corneal Stiffness in Porcine Corneas

**DOI:** 10.3390/bioengineering12121308

**Published:** 2025-11-28

**Authors:** Sophia A. Reifeltshammer, Hannah Seferovic, Malavika H. Nambiar, Philippe Büchler, Theo G. Seiler, Jascha Wendelstein, Matthias Bolz, Peter Hinterdorfer, Yoo Jin Oh, Isaak Fischinger

**Affiliations:** 1 Department of Ophthalmology and Optometry, Kepler University Clinic, 4020 Linz, Austria; 2 Department of Ophthalmology and Optometry, Johannes Kepler University, 4020 Linz, Austria; 3 Institute of Biophysics, Johannes Kepler University, 4020 Linz, Austria; 4 ARTORG Center for Biomedical Engineering Research, University of Bern, 3011 Bern, Switzerland; 5 Department of Ophthalmology, Inselspital Bern, University of Bern, 3011 Bern, Switzerland; 6 Department of Ophthalmology, Ludwig-Maximilians-University Munich, 80539 Munich, Germany; 7 Institut für Refraktive und Ophthalmo-Chirurgie (IROC), 8002 Zurich, Switzerland; 8 Augentagesklinik Spreebogen Berlin, 10559 Berlin, Germany

**Keywords:** corneal crosslinking, atomic force microscopy, uniaxial tensile test, corneal biomechanics

## Abstract

Uniaxial tensile testing and atomic force microscopy (AFM) nanoindentation experiments are two valuable methods used to quantify changes in stiffness after corneal crosslinking (CXL). Here, we apply these methods by characterizing corneal stiffness ex vivo before and after CXL. Sixty-two fresh porcine corneas were divided into three groups: an untreated control group, a CXL3 group treated with the Dresden protocol, and a CXL9 group treated with the accelerated protocol. Biomechanical testing was then performed using either uniaxial tensile testing or AFM nanoindentation. Uniaxial tensile testing revealed a significant increase in corneal stiffness for the CXL3 group compared to the control group (*p* < 0.05). At 10% strain, the CXL3 and CXL9 groups exhibited increases in stiffness of 96% and 48%, respectively, compared to the control group. In contrast, AFM analysis revealed no significant differences in stiffness, showing 28% and 16% increases in the CXL3 and CXL9 groups, respectively, compared to the control group. The results suggest that uniaxial tensile testing provides a robust, sample-averaged measure of global stiffening. Interestingly, AFM nanoindentation enables mapping of localized biomechanical changes with high spatial resolution but is less sensitive to overall biomechanical changes induced by CXL.

## 1. Introduction

Keratoconus is a progressive ectatic corneal disorder involving stromal thinning, biomechanical weakening, and conical protrusion, resulting in irregular astigmatism, reduced visual acuity, and potential vision loss. The introduction of corneal crosslinking (CXL) revolutionized the treatment of keratoconus, as the first treatment option shown to sufficiently halt the progression of keratectasia [1]. First described in 1998 by Spoerl et al. [1], the Dresden protocol became the first standardized corneal crosslinking protocol [1,2]. This protocol relies on the induction of crosslinks using riboflavin and ultraviolet-A (UV-A) irradiation and has been shown to effectively increase the stiffness of weakened corneal stroma^2,3^. Since then, many different CXL protocols have been evaluated in preclinical and clinical settings. Biomechanical effects have been quantified using inflation testing, Brillouin optical microscopy, uniaxial tensile testing, and nanoindentation experiments [2,3,4,5].

In this study, we focus on two well-established examination methods: uniaxial tensile testing [2,6] and indentation experiments performed with atomic force microscopy (AFM) [7,8,9,10]. Uniaxial tensile testing is a common method for characterizing the mechanical properties of various organic and inorganic tissues, such as blood vessels [11], tendons [12] and corneas [13]. In this technique, a specimen is stretched along one axis under a controlled varying load, while its deformation is continuously recorded. The resulting stress–strain curves provide valuable data for deriving different tissue properties, such as Young’s modulus, anisotropy, and viscoelasticity. Indentation experiments are a common AFM application for characterizing various materials, including tissue mechanobiology [14], different cell types [15,16,17], and bacterial surfaces [10]. In AFM nanoindentation, a cantilever with an attached nanoscale tip performs as a spring according to Hooke’s law. The tip is brought into contact with the sample surface and is gently pushed further into the sample, leading to an upward deflection of the cantilever during indentation. Thus, the mechanical and viscoelastic properties of the sample underneath the tip are probed. The deflection of the cantilever versus its movement is recorded, allowing to derive elastic properties and Young’s modulus through fitting the recorded deflection of the cantilever.

The effects of CXL are largely confined to the anterior stroma [3,18,19,20], and the cornea exhibits regional and depth-dependent variation in collagen lamellar orientation and interweaving across its thickness, particularly within the stroma [20]. Because of these local peculiarities and the different measurement modalities, uniaxial tensile testing and AFM nanoindentation result in measured stiffening of different orders of magnitude when investigating the effects of corneal crosslinking. AFM nanoindentation usually yields Young’s modulus values in the kilopascal (kPa) range [9,18,21,22], whereas uniaxial tensile testing yields higher megapascal (MPa) values [2,6]. This makes it challenging to compare the results of the two methods. Furthermore, differences in study design and protocol implementation complicate comparisons of the results. The lack of standardized protocols has also led to substantial variability even within the same measurement modality [2,4,8,21,23,24].

In this study, we aim to thoroughly compare uniaxial tensile testing and AFM nanoindentation, by investigating corneas treated with the same crosslinking protocol and highlighting differences in the measurement procedures, data collection, and final results. 

## 2. Materials and Methods

### 2.1. Specimen Preparation

**Figure 1:**
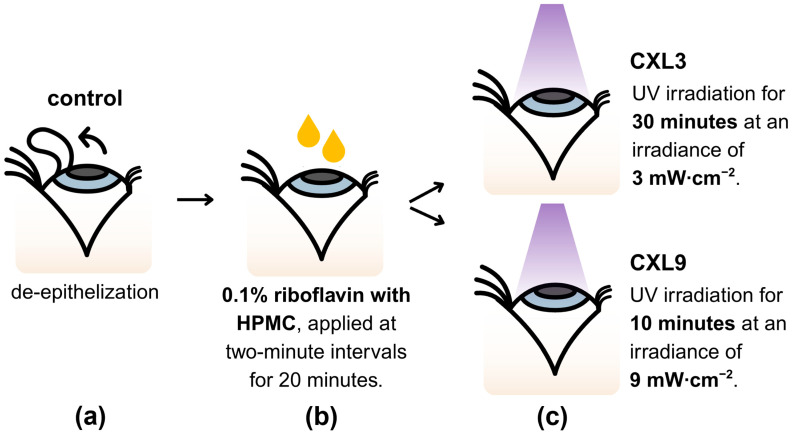
Crosslinking (CXL) procedure. (**a**) Corneal de-epithelization was performed using a hockey knife. Eyes in the control group received no additional treatment aside from immersion in a 16% dextran solution to ensure uniform hydration. (**b**) A 0.1% riboflavin solution containing hydroxypropyl methylcellulose (HPMC) was applied at two-minute intervals for 20 min, followed by (**c**) irradiation with UV-A (wavelength of 365 nm) according to the Dresden protocol (irradiance of 3 mW/cm^2^, irradiation time of 30 min, total fluence of 5.4 J/cm^2^) for the CXL3 group, and according to the accelerated protocol (irradiance of 9 mW/cm^2^, irradiation time of 10 min, total fluence of 5.4 J/cm^2^) for the CXL9 group.

Sixty-two fresh porcine eyes were collected from the local abattoir immediately after enucleation. They were inspected to ensure integrity of the globe and cornea. The eyes were then randomly divided into three groups: a control group with native corneas and no irradiation, a CXL3 group with 3 mW/cm^2^ irradiance, and a CXL9 group with 9 mW/cm^2^ irradiance (Figure 1). Each group was subsequently divided into two treatment groups: uniaxial tensile test or AFM nanoindentation.

Corneal thickness was evaluated using a pachymeter (SP-100, Tomey Corporation, Nagoy, Japan) before and after epithelial ablation. Only eyes with post-ablation pachymetry between 660 µm and 810 µm were included. De-epithelization was performed using a hockey knife.

### 2.2. Crosslinking Procedure

For the CXL3 and CXL9 groups, a 0.1% riboflavin solution containing hydroxypropyl methylcellulose (HPMC) (VibeX Rapid, Glaukos, Aliso Viejo, CA, USA) was applied to the corneal surface at two-minute intervals for 20 min after de-epithelization (Figure 1b). For the CXL3 group, the Dresden protocol was used, with an irradiance of 3 mW/cm^2^, a 30 min irradiation time, and a total fluence of 5.4 J/cm^2^ (Figure 1c, first line). The CXL9 group received irradiation according to the accelerated protocol, with an irradiance of 9 mW/cm^2^, a 10 min irradiation time, and a total fluence of 5.4 J/cm^2^ (Figure 1c, second line). According to group protocol, the eyes received irradiation with a wavelength of 365 nm (Mosaic, Glaukos, Aliso Viejo, CA, USA). After treatment, corneal discs were excised and stored in a 16% dextran solution.

### 2.3. Sample Preparation for Uniaxial Tensile Testing

**Figure 2:**
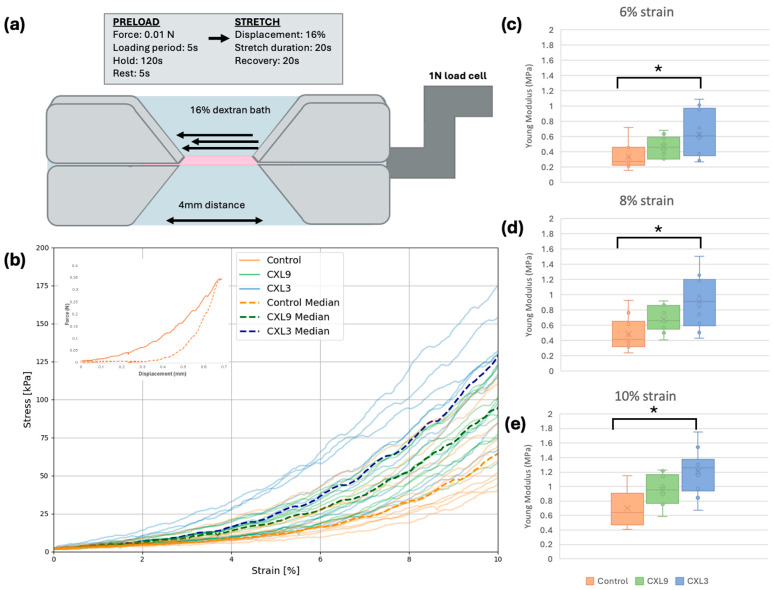
Uniaxial tensile testing for determining Young’s modulus. (**a**) Uniaxial tensile testing was conducted on corneal strips mounted at a distance of 4 mm on a 1 N load cell and immersed in 16% dextran. (**b**) Stress–strain curves were derived from the recorded force-displacement data (inset image, orange curve) and analyzed up to 10% strain. All three groups (orange = control; green = CXL9; blue = CXL3) exhibited variability, with overlapping stress–strain curves between groups. The median values for each group are indicated by dotted lines, showing an increase in stress for CXL9 and the highest increase for CXL3 compared to the control group. (**c**–**e**) Young’s modulus for each group at (**c**) 6%, (**d**) 8%, and (**e**) 10% strain. A statistically significant difference (* *p* < 0.05) was observed between the control group and the CXL3 group.

Corneal discs were cut into 5 mm wide strips along the superior-inferior axis. The samples were then mounted on a testing device equipped with a 1 N load cell (UStretch Cellscale, Waterloo, ON, Canada), with two clamps placed 4 mm apart and immersed in a 16% dextran solution to prevent drying out (Figure 2a) [6,25]. The test sequence was split into a preload and a stretch set. To ensure equal tension of the tissue, a pre-stress of 5 × 10^3^ Pa was applied to each specimen for 120 s in the preload sequence. The test sequence proceeded with a specimen displacement of 16% of the total strain magnitude for a duration of 20 s. Strain was increased at a rate of 0.035 mm/s, as described in previous studies [6,25]. The sequence concluded with a 20 s recovery period. Changes in force were recorded every 10 ms during extension. Stress and strain were calculated at each measurement point (Figure 2b), and Young’s modulus was calculated at 6%, 8% and 10% strain (Figure 2c–e).

### 2.4. Sample Preparation for AFM Nanoindentation

Similar to the spring constants used in related studies [9,20,21,22], silicon probes with a nominal spring constant of 0.5–9.5 N·m^−1^ (PtSi-FM-10, Nanosensors, Neuchatel, Switzerland) were used for the indentation experiments performed with AFM. The same cantilever, with a spring constant of 2.8 N·m^−1^ determined by the thermal noise method [26,27], was used for all measurements. Prior to the indentation experiments, the sensitivity of the cantilever (the proportionality between the output voltage of the photodetector and the deflection of the cantilever) was derived from the slope of a force-distance curve recorded on a rigid glass substrate.

**Figure 3:**
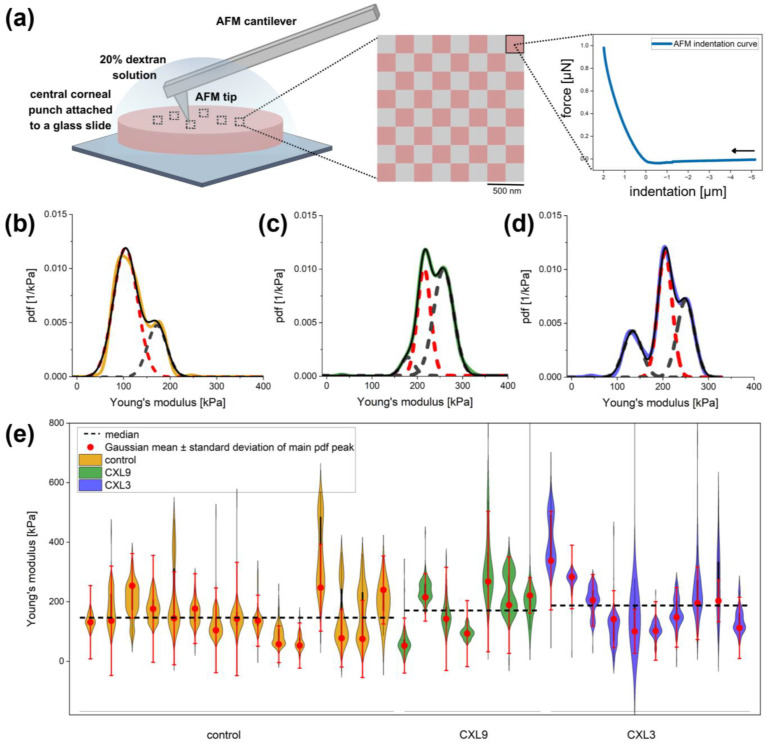
AFM nanoindentation for determining Young’s modulus. (**a**) For the AFM nanoindentation experiments, a central corneal punch was fixed to a glass slide and covered with a 16% dextran solution. Force-indentation curves were recorded by continuously moving the AFM tip towards the surface, indenting it into the corneal sample, and then retracting the tip again. Force-indentation curves were recorded at up to six different positions. At each position, 8 × 8 curves were recorded within an area of 4 µm^2^. Young’s modulus was calculated by analyzing the indentation part of the approach curve (blue curve, 0 µm to −2 µm). (**b**–**d**) Probability density functions (pdfs) of Young’s moduli of the control (yellow), CXL9 (green) and CXL3 (blue) groups were fitted with multi-Gaussian functions (red and grey). Mean and standard deviation of main peaks (red) were extracted for further analysis. (**e**) Violin plots showing the Young’s modulus data distribution for each corneal sample within the control (yellow), CXL9 (green) and CXL3 (blue) groups. The mean and standard deviation derived from the Gaussian fit of the main peak of the pdfs are plotted as red dots and error bars. The black dashed lines show the median of all data for each group.

Corneal discs were prepared by cutting a round specimen from the central cornea using an 8 mm diameter corneal punch (Vacuum Donor Cornea Punch, Barron Precision Instruments, L.L.C., Grand Rapids, MI, USA). The AFM measurements were performed on the corneal discs immersed in 20% dextran to maintain uniform hydration (Figure 3a, left image). Force-distance curves (Figure 3a, right image) were recorded in force-volume mode on an 8 × 8 grid with an area of 4 µm^2^ (Figure 3a, middle image), with approach and retraction speeds of 2 µm/s and a maximum loading force of 1.1 µN. Such force-volume measurements were recorded at up to six different positions on each corneal sample.

### 2.5. Statistical Analysis—Uniaxial Tensile Testing

For the uniaxial tensile testing, the corresponding stress *σ* was calculated using the applied force *F* and cross-section area *A* of the sample with

(1) $$\sigma =\frac{F}{A}$$

Cross-section *A* was derived by multiplying the sample width (5 mm) by the measured corneal thickness after epithelial ablation (post-ablation pachymetry). Strain values were calculated as

(2) $$\varepsilon =\frac{∆L}{L_{o}}$$

where ∆*L* is the change in length and *L_0_* is the initial length at the beginning of the measurement.

With the determined stress *σ* and strain *ε* values, Young’s modulus *E* was calculated with

(3) $$E=\frac{\sigma }{\varepsilon }$$

Furthermore, the tangent modulus was calculated by performing a linear fit around the strain of interest within a region of ± 0.3%.

Measured data were exported into MS Excel (Microsoft Corporation, Washington, DC, USA) and analyzed using SPSS (Version 29.0, International Business Machines Corporation, Armonk, NY, USA). Data was analyzed for normal distribution using Shapiro–Wilk test as well as Q-Q plots. Analysis of the Young’s modulus of the three subgroups (control, CXL3, CXL9) was performed using the Kruskal–Wallis test followed by post hoc Bonferroni correction for multiple comparisons. Differences in pachymetry were analyzed using the Kruskal–Wallis test. A *p*-value < 0.05 was defined as statistically significant.

### 2.6. Statistical Analysis—AFM Nanoindentation

For AFM measurements, Young’s modulus was calculated with the Sneddon model [28] using PicoView 1.20 software (Keysight Technologies, Santa Clara, CA, USA). The Sneddon model describes the relation between the force *F* applied by the indented AFM tip to the sample surface and the Young’s modulus *E* as

(4) $$F=\frac{2E\;\tan\left(\alpha \right)}{\pi (1-v^{2})}\delta^{2}$$

where *δ* is the deformation of the sample surface caused by the indenting tip. The calculation was performed using a tip radius *R* of 25 nm, a tip half angle *α* of 30°, and a Poisson’s ratio *ν* of 0.5 for biological samples.

Data was analyzed for normal distribution using the Shapiro–Wilk test as well as Q-Q plots. Analysis of the Young’s modulus of the three groups (control, CXL3, CXL9) was performed using the Kruskal–Wallis test followed by post hoc Bonferroni correction for multiple comparisons. Next, Young’s modulus measurements for each corneal sample were represented as an experimental probability density function (pdf) by adding up Gaussians of unitary area (Figure 3b, colored line). The means of the added-up Gaussians in the pdf were the determined Young’s modulus values, and the Gaussian widths corresponded to the estimated measurement error for Young’s modulus (10 kPa). The resulting pdf therefore comprises the Young’s modulus data and can be considered equivalent to a continuous histogram, which has a higher resolution than a conventional histogram and avoids binning artifacts. Subsequently, each pdf was fitted with the sum of Gaussians (Figure 3b, dashed Gaussian fit). With that, the mean and standard deviation of the highest peak of each pdf (Figure 3b–d, dashed red Gaussian fit, Figure 3e, red dots and error bars) was determined and were further used to calculate the average mean and standard error of each group (control, CXL9, and CXL3).

## 3. Results

### 3.1. Uniaxial Tensile Testing

Using the uniaxial tensile testing, a total of 30 eyes were analyzed: 10 from the control group, 10 from the CXL3 group and 10 from the CXL9 group. No significant differences in post-ablation pachymetry were found between the samples of all groups (*p* = 0.249).

Stress and strain were calculated at each measurement point (Figure 2b). No normal distribution of stress values at 6%, 8% or 10% strain in any of the three groups was found. All three groups showed variability, with overlapping stress–strain curves between groups. Median stress values increased in the CXL9 group, with the highest values found in the CXL3 group compared to the control group (Table 1). At 10% strain, the greatest differences in median stress were observed between the control group and the CXL9 and CXL3 groups, with increases of 48% and 99%, respectively.

Next, Young’s modulus was calculated at strains of 6%, 8% and 10% (Figure 2c–e, Table 1). Differences in Young’s modulus at 6%, 8% and 10% strain between groups were compared using Kruskal–Wallis test. At 6% strain, Kruskal–Wallis indicated a significant difference (*p* = 0.024), with subsequent testing yielding a significant difference between the control and CXL3 (*p* = 0.023, adjusted). Similarly, at 8% strain (Kruskal–Wallis, *p* = 0.007), a significant difference between the control and CXL3 was found (*p* = 0.005, adjusted), as well as at 10% strain (Kruksal-Wallis *p* = 0.002), where a significant difference between control and CXL3 was found (*p* = 0.002, adjusted). The largest differences were found at 10% strain, with calculated median (IQR) Young’s moduli of 0.644 (0.329) (control), 0.950 (0.313) MPa (CXL9), and 1.263 (0.295) MPa (CXL3), respectively (Figure 2e, Table 1).

Furthermore, tangent modulus was calculated at 6%, 8% and 10% strain (Table 1). At 10% strain, Kruskal–Wallis test indicated a significant difference between groups (*p* = 0.037); however, subsequent post hoc testing with adjusted *p*-values did not reveal significant pairwise differences between groups. At 8% strain (Kruskal–Wallis *p* = 0.05 and *p* = 0.012, respectively)), a significant difference between the control and the CXL3 group (*p* = 0.008, adjusted) as well as the control and the CXL9 group (*p* = 0.033, adjusted) was observed. 

### 3.2. AFM Nanoindentation

A total of 32 eyes were analyzed in the AFM nanoindentation experiments: 15 from the control group, 7 from the CXL9 group and 10 from the CXL3 group.

The median Young’s modulus showed an increasing tendency in the CXL9 group and an even greater tendency in the CXL3 group compared to the control group. The respective median and interquartile range (IQR) values were 146.71 (99.36) (control), 170.60 (137.21) kPa (CXL9), and 187.26 (143.08) kPa (CXL3) (Table 2). This represents an increase in Young’s modulus of around 16% and 28% for the CXL9 and CXL3 groups, respectively. The normality of the data was assessed using the Shapiro–Wilk test. As the data did not meet the normality assumption (*p* < 0.05), the non-parametric Kruskal–Wallis test was used to analyse differences between the three groups. No statistically significant differences were observed between groups (Kruskal–Wallis, *p* = 0.42). Subsequent pairwise comparisons using Bonferroni correction also confirmed that there were no significant differences between any group pairs (control vs. CXL9, adjusted *p* = 1.0; control vs. CXL3, adjusted *p* = 0.58; CXL9 vs. CXL3, adjusted *p* = 1.0).

To gain a clearer understanding of how Young’s modulus was distributed throughout each corneal sample, and to see if there were dominant, most probable values of Young’s modulus for each sample, the Young’s modulus data for each sample was summed up in an experimental probability density function (pdf) (Figure 3b–d and Figures S1–S3, colored line). Most pdfs of all groups (control, CXL9, CXL3) showed a clear dominant peak and few or no other less pronounced peaks (Figures S1–S3). These pdf peaks were fitted with sums of Gaussians, yielding multiple Young’s modulus distributions (Figure 3b–d and Figures S1–S3, dashed red and gray fits). The mean and standard deviation of the Gaussian fit for the most dominant peak of each pdf (Figure 3b, dashed red fit) were extracted for each corneal sample (Figure 3e, red dots and error bars), providing the most probable measured Young’s modulus values (Figure 3e, red dots and error bars). The Gaussian means of the most probable Young’s modulus values for each group were in good agreement with the median of all data (Table 2).

## 4. Discussion

In this study, we compared uniaxial tensile testing and nanoindentation experiments using AFM to evaluate their ability to detect biomechanical changes in the corneal stroma of porcine corneas following two different crosslinking procedures: the Dresden protocol (CXL3 group), which uses a longer irradiation time (30 min) with lower irradiance (3 mW/cm^2^) and the accelerated protocol (CXL9 group), which uses a shorter irradiation time (10 min) with higher irradiance (9 mW/cm^2^).

Uniaxial tensile testing revealed that the CXL3 group exhibited significantly greater corneal stiffness than the control group, with a 1.96-fold increase in median Young’s modulus at 10% strain (Table 3). These results align with those initially published by Wollensak et al. [2], who established the Dresden protocol and observed 1.80-fold stiffening at 8% strain using dextran-riboflavin. However, other groups have reported lower increases in stiffness, such as a 1.33-fold increase at 8% strain [19] and a 1.37-fold increase at 10% strain [24], although stiffness values were still significantly higher than those of the control group.

In our study, no significant differences were observed in uniaxial tensile testing between the accelerated protocol (CXL9) and the control group or between the accelerated protocol and the CXL3 group. Median stiffness increased by a factor of 1.48 at 10% strain compared to the control group (Table 3). Using a similar test setup to that employed in our study, Abrischamachi et al. [29] observed a comparable trend in the impact of the crosslinking protocols on corneal stiffness. The Dresden protocol resulted in greater stiffening, whereas the accelerated protocol yielded less pronounced changes in corneal stiffness. Compared to the control group, they reported increases of 1.22- and 1.33-fold for CXL9 and CXL3, respectively [29]. Investigating a larger number of corneal samples (n = 50) than in our study, Hammer et al. [24] found increases of 1.17- and 1.37-fold in the accelerated and Dresden protocols, respectively, at 10% strain with a statistically significant difference between the control group and the CXL9 and CXL3 groups.

Furthermore, we evaluated the tangent modulus, which describes the nonlinear response of the corneal tissue. Therefore, it might be a better choice than the Young’s modulus for this study. However, the comparability to literature remains limited, as the investigation of the nonlinear response of the corneal tissue is often simplified by assuming linear properties and Young’s modulus is reported. Tangent modulus was not significant at 10% strain but significant differences at 6% and 8% were found. At 10% strain, a 1.95- fold increase in median tangent modulus for the CXL3 group and a 1.69-fold increase for the CXL9 group was observed, which is comparable to the above reported changes in Young’s modulus.

In our study, AFM nanoindentation experiments revealed the same qualitative trend: The Dresden protocol (CXL3) resulted in the greatest increase in stiffness, while the accelerated protocol (CXL9) resulted in a less pronounced increase. Compared to the control group, there was an average increase in median Young’s modulus of 1.16-fold in the CXL9 group and 1.28-fold in the CXL3 group. However, group differences were not statistically significant (Table 3).

Notably, Young’s modulus varied across positions even within the same cornea in both CXL9 and CXL3 treated and untreated samples. Because AFM nanoindentation measures a local response under the tip, such variability likely reflects spatial differences in collagen lamellar orientation across the cornea [18], interweaving, as well as stromal matrix contributions [13]. In addition, we found positions on treated samples of groups CXL9 and CXL3 on which almost no stiffness increase was observed, as well as positions with a pronounced effect compared to the control group. This variation may be due to the varying diffusion of riboflavin to deeper corneal layers or to differences in oxygen concentration during treatment, both of which affect crosslinking efficiency [1,23,30,31,32]. However, looking at the most probable Young’s modulus values measured, the tendency for increased stiffness became more pronounced, with increases of 1.17-fold and 1.28-fold for the CXL9 and CXL3 groups, respectively, compared to the control group.

Other studies investigating the effect of the Dresden protocol with AFM nanoindentation on human corneas showed large variability in stiffness increases, ranging from 1.70-fold [8], 1.90-fold [18] and 5.57-fold [21] increases compared to the control group. Dias et al. [4] evaluated corneal stiffness in porcine corneas using the Dresden protocol and the accelerated protocol. They found a statistically significant difference for the anterior stroma between the Dresden protocol and the control group, with a 2.63-fold increase in Young’s modulus. The accelerated protocol showed a 1.43-fold increase compared to the control group. However, in contrast to our study where Young’s modulus was measured on distinct positions, in this study it was only measured at one position on the corneal sample, resulting in smaller Young’s modulus variations [4].

The measured Young’s modulus values differed between the two techniques used, with higher values obtained by uniaxial tensile testing. Others [13] have also observed this phenomenon and have proposed that it may be because the strength of the collagen bundles primarily contributes to the measured forces in the uniaxial tensile test. These forces are orders of magnitude higher in tension than in compression [13]. In AFM nanoindentation, however, the tip indents vertically into the corneal surface, compressing the area beneath it. Thus, the response of the stromal matrix, rather than the properties of the collagen fibers only, is characterized [13].

Although we have followed well-established measurement protocols and the results of the two measurement methods are consistent with prior reports, several limitations should be noted. Test setups and protocols vary across literature, so variability in stiffness obtained in our study and in others can be attributed to factors including differences in sample sizes, dextran solutions, mechanical testing devices, and measurement protocols. Future studies are needed to establish a testing standard that will help make results more comparable across different studies.

In conclusion, both the uniaxial tensile testing and the AFM nanoindentation experiment revealed the same overall trend: an increase in average stiffness in corneas treated with the accelerated protocol (CXL9 group) and the greatest increase observed in corneas treated with the Dresden protocol (CXL3 group). However, the differences between the groups were more pronounced in the uniaxial tensile testing. A significant difference was found between the CXL3 group and the control group. Uniaxial tensile testing measures average stiffness changes across the entire corneal sample. In contrast, AFM nanoindentation offers a localized assessment that captures spatial heterogeneity across the cornea. This feature might be beneficial for detecting local biomechanical changes, as it might occur after customized CXL, in which different fluences are applied throughout the treated cornea. On the other hand, this can lead to broader distributions of Young’s modulus, making data interpretation more challenging and potentially reducing measurement sensitivity. Accordingly, the two approaches are complementary. The uniaxial tensile testing provides a robust, sample-averaged measure of global stiffening, whereas AFM nanoindentation enables mapping of localized biomechanical changes at higher spatial resolution. Together, they offer a comprehensive evaluation of CXL-induced corneal stiffening.

## Figures and Tables

**Table 1:** Stress (median (IQR)), calculated Young’s modulus (median (IQR)) and tangent modulus (median (IQR)) at 6%, 8% and 10% strain for control, CXL9 and CXL3 group measured with uniaxial tensile test.

	Stress [kPa]			Young’s Modulus [MPa]			Tangent Modulus [MPa]		
	Control (n = 10)	CXL9 (n = 10)	CXL3 (n = 10)	Control (n = 10)	CXL9 (n = 10)	CXL3 (n = 10)	Control (n = 10)	CXL9 (n = 10)	CXL3 (n = 10)
**6% strain**	16.34 (11.18)	27.46 (13.68)	36.58 (35.09)	0.273 (0.185)	0.458 (0.240)	0.609 (0.521)	0.475 (0.702)	1.152 (0.509)	1.624 (1.027)
**8% strain**	33.25 (33.04)	51.93 (21.77)	72.06 (41.89)	0.415 (0.247)	0.661 (0.267)	0.911 (0.477)	0.976 (0.841)	2.130 (0.877)	2.429 (1.435)
**10% strain**	64.38 (33.04)	95.05 (31.34)	128.23 (29.88)	0.644 (0.329)	0.950 (0.313)	1.263 (0.295)	1.625 (1.251)	2.741 (1.392)	3.175 (1.916)

**Table 2:** Young’s modulus (median (IQR)) of all data and Young’s modulus (mean ± standard error) of mean and standard deviation derived from Gaussian fitting for control, CXL9 and CXL3 group measured with AFM nanoindentation.

Young’s Modulus [kPa] (Median (IQR))			Young’s Modulus [kPa] (Average of Means ± Standard Error Derived from Gaussian Fits)		
control (n = 15)	CXL9 (n = 7)	CXL3 (n = 10)	control (n = 15)	CXL9 (n = 7)	CXL3 (n = 10)
146.71 (99.36)	170.60 (137.21)	187.26 (143.08)	143.37 ± 14.57	168.68 ± 22.92	182.82 ± 14.16

**Table 3:** Summary of the median and interquartile range (IQR) of Young’s modulus measured across groups. Fold changes were calculated as the median ratio relative to the control group.

Uniaxial Tensile Testing (10% Strain)			AFM Nanoindentation		
Group	Median (IQR) [MPa]	Fold-Change (Versus Control)	Group	Median (IQR) [MPa]	Fold-Change (Versus Control)
control	0.644 (0.329)	1.00	control	0.147 (0.099)	1.00
CXL9	0.950 (0.313)	1.48	CXL9	0.171 (0.137)	1.16
CXL3	1.263 (0.295)	1.96	CXL3	0.187 (0.143)	1.28

## Data Availability

Data presented in this article is available on request from the corresponding authors.

## Supplementary Materials

The following supporting information can be downloaded at: https://www.mdpi.com/article/10.3390/bioengineering12121308/s1, Figure S1: Experimental probability density functions of corneal samples in the control group; Figure S2: Experimental probability density functions of corneal samples in the CXL9 group; Figure S3: Experimental probability density functions of corneal samples in the CXL3 group.

## Abbreviations

The following abbreviations are used in this manuscript:

AFM	Atomic force microscopy
CXL	Corneal crosslinking
HPMC	Hydroxypropyl methylcellulose
UV-A	Ultraviolet-A
