# Supplementary material for: Comparative Analysis of Two Measurement Modalities for Ex Vivo Analysis of Corneal Stiffness in Porcine Corneas
Source: Bioengineering (Basel). 2025 Nov 28;12(12):1308. doi: 10.3390/bioengineering12121308 (PMC12729501; doi:10.3390/bioengineering12121308)
Supplement: Supplementary file 1 [file bioengineering-12-01308-s001.zip › bioengineering-3972446-supplementary.pdf]

Supplementary Material

of the manuscript

Comparative analysis of two measurement modalities for ex vivo analysis of corneal stiffness in porcine corneas

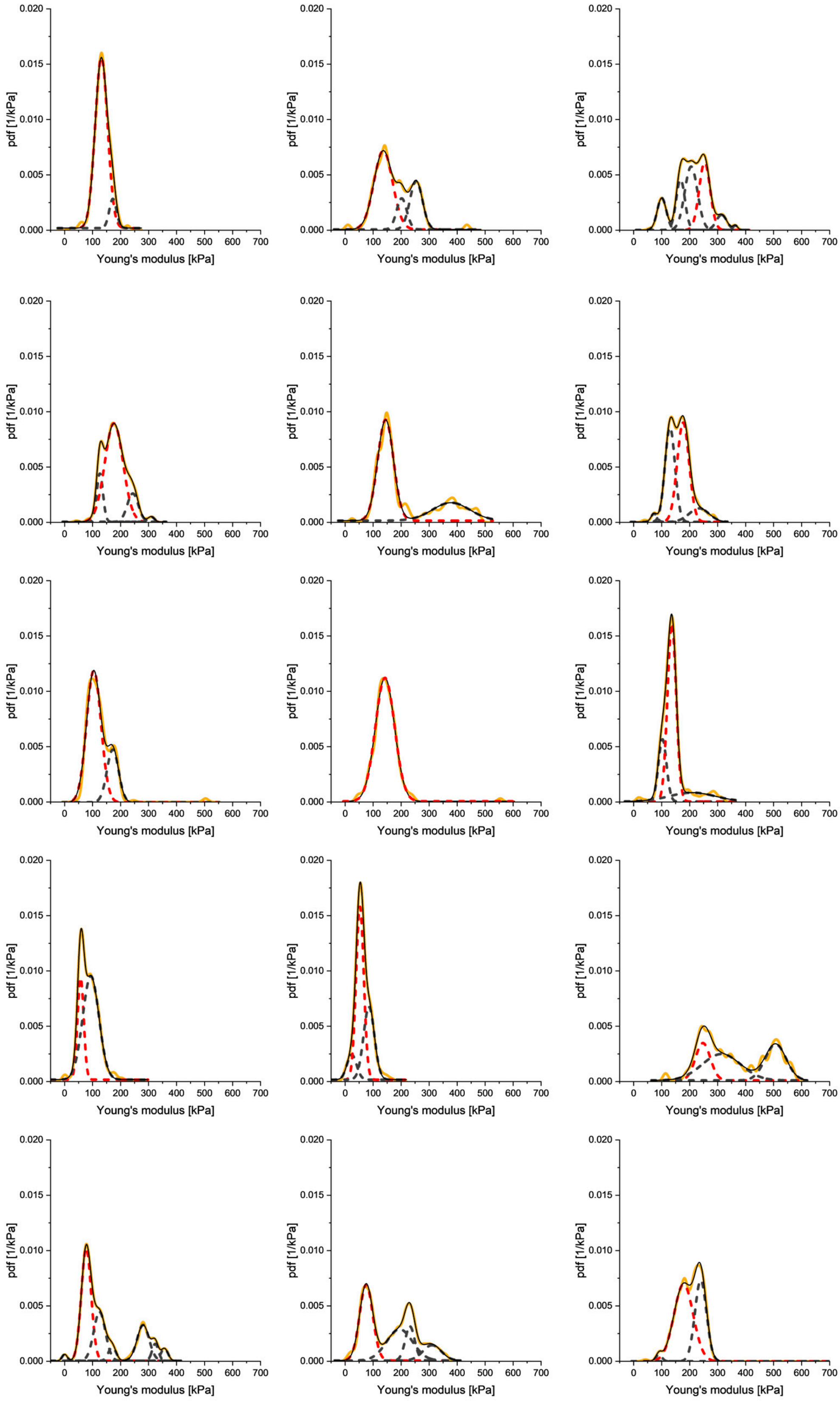

Figure S1 | Experimental probability density functions (pdfs, yellow curves) of corneal samples in the control group. The peaks were fitted with multiple Gaussians (red and grey dashed lines), highlighting different populations within the determined Young's modulus of the sample. Main peaks are highlighted in red.

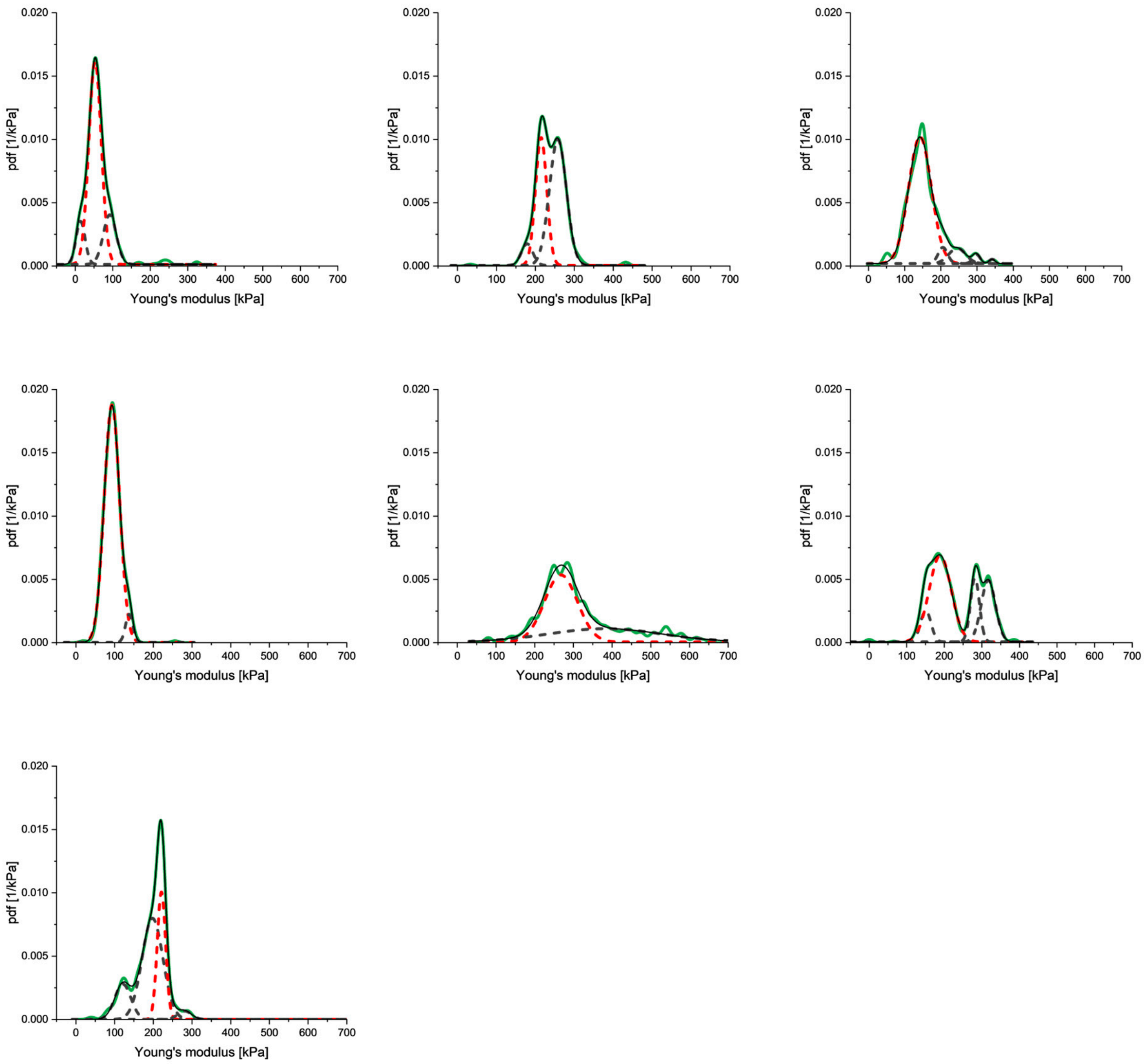

Figure S2 | Experimental probability density functions (pdfs, green curves) of corneal samples in the CXL9 group. The peaks were fitted with multiple Gaussians (red and grey dashed lines), highlighting different populations within the determined Young's modulus of the sample. Main peaks are highlighted in red.

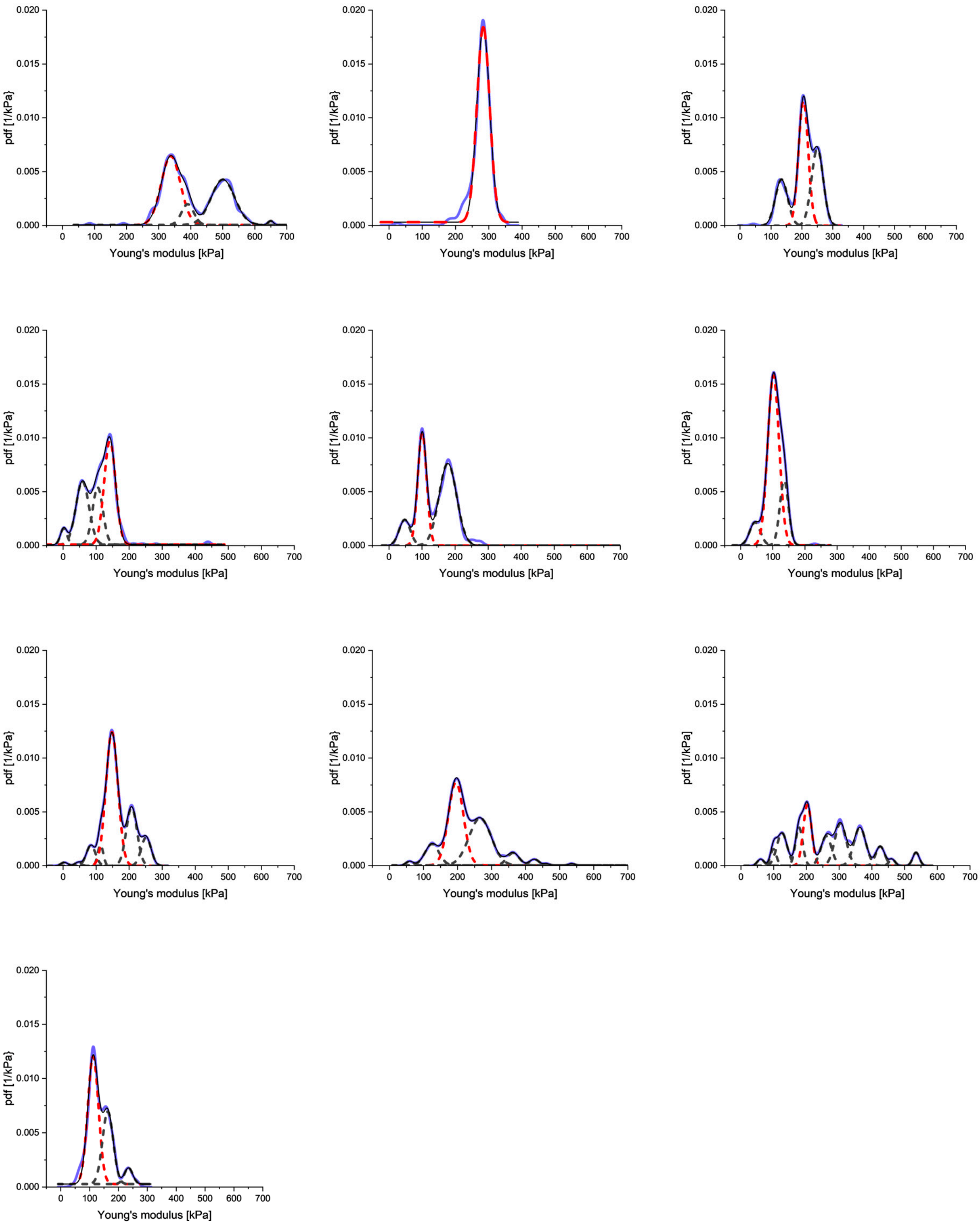

Figure S3 | Experimental probability density functions (pdfs, blue curves) of corneal samples in the CXL3 group. The peaks were fitted with multiple Gaussians (red and grey dashed lines), highlighting different populations within the determined Young’s modulus of the sample. Main peaks are highlighted in red.
